# The evaluation of the three edible tissues of dead adult Chinese mitten crabs (*Eriocheir sinensis*) freshness in harvest season, based on the analysis of TVBN and biogenic amine

**DOI:** 10.1186/s40064-016-3434-4

**Published:** 2016-11-03

**Authors:** Xiaozhen Yang, Jinbiao Zhang, Yongxu Cheng

**Affiliations:** Key Laboratory of Genetic Resources for Freshwater Aquaculture and Fisheries, Shanghai Ocean University, Shanghai, 201306 China

**Keywords:** Chinese mitten crab, Total volatile base nitrogen test, Biogenic amines, Freshness, Food safety

## Abstract

This study was carried out to evaluate the quality of the three edible tissues (muscles, hepatopancreas and gonads) of the male and female Chinese mitten crab (*Eriocheir sinensis*) in natural harvest season, based on total volatile base nitrogen test (TVBN) and biogenic amines analysis at intervals time of 3 h extended for 24 h during 30 °C storage (a mean ambient temperature in harvest season). In addition, the relationship between the value of TVBN and storage time or main biogenic amine was evaluated. Results showed that TVBN level of female gonad reached the recommended limit (30 mg/100 g) at 3 h, and the times of reaching the recommended limit were 15 h in both sexes muscles, while in male and female hepatopancreas reached the recommended limit at times of over 24 and 24 h respectively. Moreover, putrescine was highest in amount in all biogenic amines analyzed. We suggested that TVBN value could be as a good indicator for Chinese mitten crab freshness. According to analysis for biogenic amine accumulated in edible tissues of crab, it was possible that dead crab poisoning may be caused by putrescine. Dead Chinese mitten crabs for over 24 h, should be not recommended for eating or processing.

## Background

The Chinese mitten crab (*Eriocheir sinensis*) is a traditional seafood source in China, where it supports an important aquaculture industry yielding high annual production, worth approximately $1.25 billion (Veilleux and de Lafontaine 2007; Cheng et al. 2008). The muscles and hepatopancreas and gonads are the most prized parts of the crab. These tissues are also usually considered as edible tissues of Chinese mitten crabs. The preferred crabs are captured during the fall, and are autumn delicacy in Shanghai cuisine and eastern China, as they have full gonads prior to reproduction (Cheng et al. 2008). The average ambient temperature in this season is 30 °C, which is highest temperature among the average of season temperature in the whole year. Chinese mitten crabs are usually cooked alive, which are similar to cooking Lobsters. However, the edible tissues of Chinese mitten crabs collected at autumn are often processed and stored in order to meeting the need of human consummation at any time. Although, these crabs (normal adult crabs and precocious crabs) collected in harvest season were generally processed after cooking, but uncooked crabs could also be processed (Gillespie et al. 1983). Moreover, for acquiring high profit, dead and deteriorating crabs were possibly also collected for processing by processors. Therefore, it is very important to evaluate the quality of crab in order to reduce the risk of eating crab.

At the present, there are two main methods of assessing seafood quality to determine its freshness. These are sensory and non-sensory methods. Sensory methods rely mostly on appearance, odors, texture and taste of the seafood whether to be acceptable or rejected while non-sensory methods use physical, biochemical, chemical and microbiological means (Huss 1995). Among the non-sensory methods, total volatile nitrogen (TVBN) test is known as an important and popular test method for evaluating various seafood freshness, such as crab, fish and mullet etc., and is well correlated with sensory analysis (Zamir et al. 1998; Özogul and Özogul 2000; Özyurt et al. 2009; Zhang et al. 2012). For example, Özyurt et al. (2009) found that TVBN values of red mullet (*Mullus barbatus*) and goldband goatfish (*Upeneus moluccensis*) were well correlated with sensory analysis.

TVBN is mainly composed of ammonia and primary, secondary and tertiary amines. The TVBN content of sea food increases as putrefaction progresses since ammonia is produced during storage as a result of the deamination of amino acids. Min et al. (2007) has also confirmed the quantities of biogenic amines (BAs) concentration are highly correlated with the TVBN concentration in beef, pork, and chicken during storage. So far it has been accepted that the quantities of BAs are also to be considered as a marker of the level of microbiological contamination in food (Vinci and Antonelli 2002a, b; Min et al. 2004a). BAs are organic compounds with a low molecular weight that are formed through the enzymatic decarboxylation of specific amino acids in various foods during storage (Hernandez-Jover et al. 1996; Min et al. 2004b). Especially, BAs are produced in foods, where high levels of protein are present, for example in seafood (Vinci and Antonelli 2002a, b). The BAs that are often found in foods including putrescine (PUT), cadaverine (CAD), histamine (HIM), tyramine (TYM), serotonin (SER), spermine (SPM) and spermidine (SPD). PUT is formed from ornithine, CAD from lysine, HIM from histidine, TYM from tyrosine, SER from TRM, SPM from PUT and SPD from SPM (Halasz et al. 1994; Chen et al. 2002). In addition, dopamine (DA) and octopamine(OA) have been found in spoilage sea food (Özyurt et al. 2009; Hernandez-Jover et al. 1996). Among BAs, HIM has been more frequently implicated in food poisoning, and PUT and CAD are known to enhance histamine poisoning (Chen et al. 2002). Histamine scrombroid/toxicity accounted for 35% of the documented seafood-associated outbreaks (Center for Science in the Public Interest 2009). Therefore, although there are no any reports about analysis of biogenic amine of dead crab and deteriorating crab collected at autumn, people commonly considered that an amount of histamine contained in dead crab and deteriorating crab, and which eventually resulted in the people fear of eating them.

In the current study, it is the first time to evaluate the quality of the edible of both the male and female Chinese mitten crab in 24 h after dead at 30 °C based on TVBN test. Moreover, we have further investigated the changes of biogenic amine of Chinese mitten crab edible tissues during 24 h of storage, and the relationship between the value of TVBN and storage time or main biogenic amine was evaluated.

## Methods

### Sample preparation

The adult normal Chinese mitten crabs were collected from the Aquaculture Technology Extension Station in Shanghai Chongming (China), and were immediately brought back to the laboratory. The average of carapace length, carapace width and body weight were 59.92 ± 6.00, 64.25 ± 2.02 mm and 122.72 ± 14.63 g for male respectively, and 57.11 ± 6.32, 60.12 ± 3.41 mm and 95.53 ± 15.14 g for female respectively. On arrival, the crabs were executed by −80 °C refrigerator storage for 2 h. Each dead crab was individually packaged by plastic bag, and all of them were stored in a cabinet with the temperature controlled at 30 ± 1 °C. The three edible tissues of crab including muscle (the whole body muscle), hepatopancreas and gonad were collected for future analysis at intervals time of 3 h extended for 24 h. Each analysis was performed in triplicate.

### Determination of total volatile base nitrogen (TVBN)

TVBN was determined with a Kjeltec System 2300 (Foss Tecator, USA) (AOAC 1995). Bases from the weighed samples were distilled in the presence of magnesium oxide. The distillate was collected in a solution of excess boric acid in the presence of indicators (methyl red mixed with bromocresol green) and titrated with 0.1 N hydrochloric acid. The TVBN value was expressed as mg N/100 g sample as described by Xing et al. (2012).

### Biogenic amine analysis

Biogenic amine analysis was carried out according to Min et al. (2007) and Ding et al. (2006). Two grams of the sample was weighed into a 50 mL polypropylene conical tube and homogenized in 10 mL of 0.4 M perchloric acid. The homogenized sample was centrifuged for 10 min at 3000 rpm and rinsed with supernatant into a 25 mL bottle through filter paper. The extraction was repeated with 10 mL of 0.4 M perchloric acid solution, mixed thoroughly with a vortex mixer and centrifuged as above. Supernatants were combined and adjusted to 25 ml with 0.4 M perchloric acid solution.

The alkalinity of a 1 mL sample extract was adjusted using 100 μL of 2 mol/L sodium hydroxide solution. A 300 μL saturated sodium bicarbonate was added as a buffer. A 2 mL dansyl chloride solution (10 mg/mL acetone) was added and incubated at 40 °C for 45 min in the dark. Residual dansyl chloride was removed by adding 100 μL ammonia (25%). After 30 min, dansylated extract was adjusted to 5 mL with 0.1 mol/L ammonium acetate/acetonitrile (1:1), and filtered through a 0.45 μm syringe filter. Ten microliters of a filtered sample was injected to the HPLC with a diode array detector (LC-10Avp series; Shimadzu, Kyoto, Japan) equipped with a C18 column (4.6 × 150 mm,shim-pack, Shimadzu, Kyoto, Japan). Gradient elution program was used with a mixture of 0.1 M ammonium acetate as solvent A and acetonitrile as solvent B. Both solvents were vacuum filtered by a membrane filter (47 mm PTFE 0.45 μm, Shanghai ANPEL Co., Ltd.) and degassed with an ultra-sonicator. The flow rate was 1 mL/min. The order of the gradient was shown as follows: 45% solvent A and 55% solvent B for 0–7 min, 50% solvent A and 50% solvent B for 7–25 min, 10% solvent A and 90% solvent B for 25–35 min, 45% solvent A and 55% solvent B for 35–45 min. The column temperature was 40 °C. The amounts of the dansyl derivatives of the biogenic amines were quantified by a measurement of the UV-absorption at 254 nm.

### Statistical analysis

Statistical analysis was performed using the SPSS software (Chicago, USA; Version 17.0). The data of relative expression of the gene which was run in different duplications and levels were used in the statistical analysis. Data represented the mean ± S.E. Statistical significance was determined using one-way analysis of variance and post hoc Duncan multiple range tests. *P* < 0.05 indicated statistical significance. Pearson’s correlation coefficients were used to determine the relationship between BAs and TVBN.

## Results and discussion

**Fig. 1:**
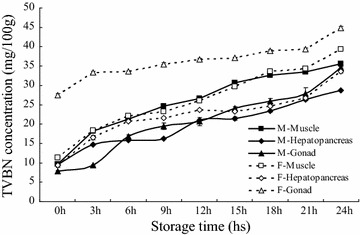
Changes in TVBN value of muscle, hepatopancreas and gonad of the Chinese mitten crab (*Eriocheir sinensis*) during storage at 30 °C

TVBN values can be used as an indicator of fitness for food consumption (Vinci and Antonelli 2002a, b). The present study was carried out to investigate and to determine the quality of the dead adult Chinese mitten crab at intervals time of 3 h extended for 24 h at 30 °C storage. The TVBN values of three edible tissues of the Chinese mitten crab for male and female, including muscle, hepatopancreas and gonad, were presented in Fig. 1. The values for TVBN increased as storage time progressed in all tissues (*P* < 0.05) (Fig. 1). This result generally agree with previous research by Zamir et al. (1998), which showed the TVBN value of Mud Crab *Scylla serrata* meat at refrigerator temperature (7 ± 2 °C) was significantly raised, approximately 12–17 mg TVBN increased at each day of storage during 7 days. The maximum recommended limit set of TVBN was not exceed 30 mg/100 g by international organization Codex Aliment Aries, EU Stander and regulators countries (Huss 1995). That meaning that seafood will be spoilage when the TVBN value researched over 30 mg/100 g. Results in the present study revealed that there was no significant (*P* > 0.05) difference for the time of TVBN value reaching recommended limit between female and male muscle (about 15 h), whereas obvious difference for the time of TVBN value reaching recommended limit were observed in gonad and hepatopancreas between female and male. The TVBN value of hepatopancreas and gonad progressively increased to 28.72 ± 2.07 mg/100 g (24 h) and 34.61 ± 1.22 mg/100 g (24 h) respectively for male, and 33.55 ± 1.38 mg/100 g (24 h) and 32.34 ± 1.31 mg/100 g (3 h) respectively for female. The times of TVBN value reached the recommended in hepatopancreas and gonad of were over 24 and 24 h respectively for male, and 24 and 3 h for female. Compared to other tissues in this study, the time of reaching recommended limit was earliest in female gonad only for 3 h, which may be due to rich proteins in gonad (Barrento et al. 2009). Many previous results have demonstrated that there are different TVBN levels in different tissues for the same species. For example, Özoğul and Özoğul (2000) reported TVBN in the belly flesh sample was the highest, whereas the lowest TVBN value was obtained with the dorsal flesh in rainbow trout (*Oncorhynchus mykiss*). In terms of the analysis of TVBN value, we suggested three edible tissues of both sexes of Chinese mitten crab shouldn’t be used for food after dead for over 24 h at higher temperature season. A high correlation was also found between TVBN and storage time (Table 1). As the TVBN value used as a freshness indicator of other animals, such as beef, pork, chicken, fish and shrimp etc., we thought that it could also be considered to be reliable indicators of the freshness of Chinese mitten crab.

**Table 1:** Pearson’s correlation coefficients between volatile basic nitrogen time and PUT concentration versus TVBN

	TVBN		
	Muscles ♂ (♀)	Hepatopancreas ♂ (♀)	Gonads ♂ (♀)
Time	0.97 (0.99)	0.99 (0.94)	0.98 (0.95)
PUT	0.90 (0.94)	0.69 (0.24)	0.95 (0.88)

This study found that the TVBN contents of muscle and hepatopancreas of both male and female, and male gonad at 0 h were generally in the range of 7.9–9.63 mg/100 g. However, the TVBN content of female gonad (27.50 mg/100 g) was higher than that of other tissues on day 0. Besides rich proteins, the higher beginning value of TVBN may be also one of the reasons that the value of TVBN value for female gonad was earliest reaching recommended limit, compared to other tissues. Chomnawang et al. (2007) have reported that TVBN content of hybrid catfish fillet samples on day 0 at 4 °C was in the range of 15.73 and 16.91 mg/100 g. Gormley et al. (2003) found that TVBN of raw whiting fillet, mackerel fillet and salmon portions chilled on day 0 at 4 °C was 13.7, 15.9 and 17.0 mg/100 g respectively. Yamanaka (1990) reported that TVBN of saury pike stored at 5 °C was 5.5 mg/100 g during storage period on day 0. These results revealed that the beginning contents of TVBN were different in different animals and tissues. Moreover, above researchers suggested that the higher levels of TVBN in muscles at day 0 of storage may be related to fish muscle undergoing some deterioration during handling (Benner et al. 2003). However, for female gonad, the higher TVBN values at 0 h might be referring to handing or biological enzymes activity. Further researches are needed to explain the reason of the higher TVBN values at 0 h.

**Fig. 2:**
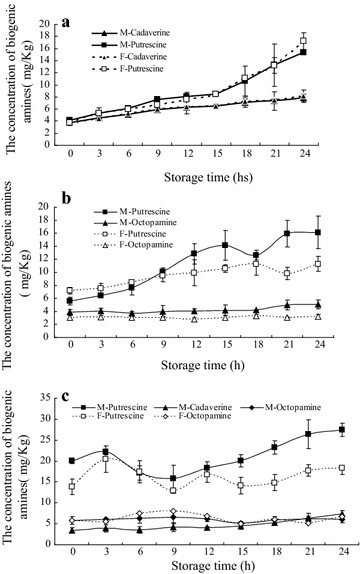
Changes in biogenic amines of muscle **a**, hepatopancreas **b** and gonad **c** of the Chinese mitten crab (*Eriocheir sinensis*) during storage at 30 °C

Figure 2 shows the changes of biogenic amines content (BAs) contents in muscles, hepatopancreas and gonads of both male and female during 24 h of storage. Although HIM from seafood often results in various food borne poisoning, and HIM in our previous study has been detected by the same analysis method in *Collichthys lucidus* and *Harpodon nehereus* muscle at same storage condition (30 °C for 24 h) (Zhang et al. 2012). However no histamine was detected in three edible tissues of dead Chinese mitten crab in the current study. Interestingly, Xu et al. (2009) found that HIM could be detected in dead Chinese mitten crab muscle. In Xu’s experiment (2009), the level of HIM in muscle was found to be 91 mg/kg at 4 °C for 72 h and 181 mg/kg 20 °C for 24 h. The following reasons can be used to explain the differences between the results of our study and Xu’s study (2009). Firstly, our study specifically analyzed adult crab and whole body muscle samples, while the development stage of crab and origin of muscle samples analyzed in Xu’s study (2009) is uncertain. At autumn, both normal adult and precocious crabs could be available in market, and their different protein organizations may possibly produce different biogenic amines after death (Wu et al. 2010). In addition, histamine is not uniformly distributed in decomposed seafood. For example, if 5 mg/100 g is found in one section, there is a possibility that other units may exceed 50 mg/100 g (Lehane and Olley 2000). Secondly, according to sample preparation from Xu’s study (2009), dead crabs were acquired by storing live ones in refrigerator (4 °C) until death, but crabs in our study were immediately executed by −80 °C refrigerator storage for 2 h, which resulted in the initial levels of TVBN and biogenic amine in our study were lower than Xu’ study. We know tied crab can stay alive at 4 °C for at least 2 weeks. The degree of freshness should be reduced during execution due to the increase of bacterial spoilage (Robson et al. 2007), which possibly results in histamine accumulation in tissues. Moreover, due to the fear of consuming time and money, most consumers or processors usually don’t prefer keeping live crab in the refrigerator for extended periods of time before cooking or processing. In general, the different samples and treatment methods for the crab could account for differences in results this study between that of Xu’ study. Moreover, many scientists have proved that the histamine level were different among different types of seafood, and associated with either storage time, temperature conditions or food technologies; for example the histamine levels were 10.0 mg/kg for Skipjack tuna at 25 °C for 10 h (Staruszkiewicz et al. 2004) and 2240.0 mg/kg for Sailfish at 25 °C for 24 h (Tsai et al. 2005). Meanwhile, such results may be explained by differences in the composition and the level of microorganisms in the seafood. In addition, some studies suggested that histamine was not always present in every “decomposed” sample, and it may be that the PUT and CAD, which give a decomposed piece of seafood its distinct putrid smell, are better markers of decomposition, and may even potentate the deleterious health effects of consuming HIM-spoiled seafood (Lehane and Olley 2000; Marks and Anderson 2005). Based on these results, we suggest that the Chinese mitten crab poisoning may be the result of PUT accumulation. As for whether or not HIM is involved in this danger, it would need to be judged by a series of detailed analyses. HIM levels in dead crab may be correlated with sample type, storage conditions, and various environmental microflora.

Among the nine biogenic amines, only PUT, CAD and OA were measured in crab edible tissues. In addition, the types of biogenic amines measured and their levels were different among the kinds of tissues. PUT could be measured in all tissues. CAD could only be found in male muscle and hepatopancreas, and female muscle, whereas OA could be found in hepatopancreas and gonad. So far, using the quantity of BAs as an index for the freshness of meat has been tried by many researchers. Mietz and Karmas (1977) proposed a sum of concentrations of HIM, PUT, CAD, SPM and SPD could be as an index of freshness for canned tuna. Later, Veciana-Nogues et al. (1997) reported that the TYM content increased significantly during tuna storage and the authors suggested that TYM content was the sum of the concentrations of HIM, CAD, TYM and PUT. Min et al. (2007) have confirmed that the content of PUT, CAD, and TYM could be as a good index for evaluating fresh beef, pork, and chicken meat freshness during storage. All of above results demonstrated that PUT and CAD were common biogenic amines, and they could be an index for evaluating freshness for various food products. Similarly, Benner et al. (2003) also found PUT and CAD are suitable for indicating decomposition in penaeid shrimp, especially for PUT. The present study, only PUT was measured in all tissues and was always higher in amount than other biogenic amines. For example, the average of PUT levels in male and female muscle, hepatopancreas and gonad at 24 h was 16.28 μg/g, 22.88 and 13.7 μg/g respectively. In comparison to that, the average amount of CAD in both sexes muscle at 24 h was 8.01 μg/g (Fig. 2a). In the hepatopancreas, the average content of CAD in male and female was 6.18 μg/g, and that of OA was 6.36 μg/g (Fig. 2b). In the gonads of both males and females, the average amount of OA was only 4.15 μg/g (Fig. 2c). Figure 2a shows that only Put and CAD could be measured in muscle. As storage time increased, the PUT levels in both male and female increased from 4.16 and 3.78 (0 h) to 15.33 and 17.22 μg/g (24 h) respectively. Compared to the 0 h, the significant increase of PUT concentration in male and female occurred on 18 and 6 h respectively (*p* < 0.05). However, the CAD levels of both male and female significant increase could be found at 12 and 6 h respectively. In the male hepatopancreas (Fig. 2b), the concentration of PUT and CAD significant increased at 24 and 21 h respectively (*p* < 0.05). The levels of PUT in both male and female gonads obviously increased with storage time increased, but significant increase was found at 12 h for male and 18 h for female (Fig. 2c). These PUT and CAD data generally agree with previous research by Shakila et al. (1995), which showed overall increases in PUT and CAD concentrations in Indian shrimp (*Parapenaeopis stylifera*) stored at 0, 5 and 30 °C. Benner et al. (2003) study showed that decomposition of the shrimp progressed more rapidly with time at progressively higher temperatures. Compared to the 0 and 12 °C, the times of PUT and CAD significant increase were earlier in higher temperatures (24 and 36 °C). This study also found that OA could be found in hepatopancreas and gonads of both sexes. However, there are no significant differences in OA levels among different storage time for both male and female (*p* > 0.05) (Fig. 2b, c).

In addition, the relationship between the value of TVBN and storage time or PUT was evaluated. In all organs, a high correlation was found between TVBN and storage time (Table 1). Pearson’s correlation coefficients of TVBN in all organs with time were higher than 0.94. Meanwhile, PUTs in muscle and gonad of both sexes and male hepatopancreas were positively correlated with TVBN (Pearson’s correlation coefficients >0.60) (Table 1). As TVBN, the PUT could also be as an indicator for decomposition in above organs of dead Chinese mitten crab, especially muscles and gonads.

Because biogenic amines as heat stable compounds have been reported, cooking or prolonged exposure to heat will not eliminate the toxin (Tapingkae et al. 2010). We suggested that biogenic amine, especially for PUT should be the most comprehensive chemical indicator for decomposition in dead Chinese mitten crab.

## Conclusion

Based on the analysis of TVBN, we suggested three edible tissues of both sexes of Chinese mitten crab shouldn’t be used for food after dead for over 24 h at higher temperature. In all of the edible tissues, the female gonad was earliest decomposition tissue compare to other tissues at higher temperatures. TVBN value could be as a good indicator for Chinese mitten crab freshness. According to analysis of biogenic amine, we suggested that the edible tissues of dead adult Chinese mitten crab poisoning may be the result of PUT accumulation. As for whether HIM involved in this dangerous, it would need be judged by a serials of details analysis, such as sample type, storage condition, and various environmental microflora.
